# Genetically engineered mouse models of head and neck cancers

**DOI:** 10.1038/s41388-023-02783-7

**Published:** 2023-07-20

**Authors:** Jason Tasoulas, Sonal Srivastava, Xiaonan Xu, Valentina Tarasova, Anastasios Maniakas, Florian A. Karreth, Antonio L. Amelio

**Affiliations:** 1 Department of Otolaryngology—Head and Neck Surgery, The University of North Carolina at Chapel Hill, Chapel Hill, NC USA; 2 Lineberger Comprehensive Cancer Center, The University of North Carolina at Chapel Hill, Chapel Hill, NC USA; 3 Department of Tumor Biology, H. Lee Moffitt Cancer Center and Research Institute, Tampa, FL USA; 4 Department of Molecular Oncology, H. Lee Moffitt Cancer Center and Research Institute, Tampa, FL USA; 5 Department of Head and Neck-Endocrine Oncology, H. Lee Moffitt Cancer Center and Research Institute, Tampa, FL USA; 6 Department of Head and Neck Surgery, The University of Texas MD Anderson Cancer Center, Houston, TX USA

**Keywords:** Cancer models, Oral cancer

## Abstract

The head and neck region is one of the anatomic sites commonly afflicted by cancer, with ~1.5 million new diagnoses reported worldwide in 2020 alone. Remarkable progress has been made in understanding the underlying disease mechanisms, personalizing care based on each tumor’s individual molecular characteristics, and even therapeutically exploiting the inherent vulnerabilities of these neoplasms. In this regard, genetically engineered mouse models (GEMMs) have played an instrumental role. While progress in the development of GEMMs has been slower than in other major cancer types, several GEMMs are now available that recapitulate most of the heterogeneous characteristics of head and neck cancers such as the tumor microenvironment. Different approaches have been employed in GEMM development and implementation, though each can generally recapitulate only certain disease aspects. As a result, appropriate model selection is essential for addressing specific research questions. In this review, we present an overview of all currently available head and neck cancer GEMMs, encompassing models for head and neck squamous cell carcinoma, nasopharyngeal carcinoma, and salivary and thyroid gland carcinomas.

## Introduction

Head and neck cancers (HNC) include squamous cell carcinomas (HNSCC) arising from oral, oropharyngeal, nasopharyngeal, hypopharyngeal, and laryngeal epithelia in addition to adenocarcinomas arising from the salivary gland carcinomas (SGC) and thyroid cancers (TC) glands. Despite significant differences in incidence across different countries [1], HNSCC is the most common malignancy of the head and neck [2] and the sixth most common cancer overall with ~878,000 new diagnoses globally in 2020 [3]. Variations in global HNSCC burden reflect differences in exposure to known risk factors including smoking and tobacco use [4], alcohol consumption [5], betel nut chewing [6], oncogenic human papillomavirus (HPV) infection [7], and low socioeconomic status [8, 9]. However, there has been a shift in the pattern of risk factors that contribute to HNSCC development in recent decades. Most notably, smoking and tobacco use have been declining [10] and, consequently, the number of HNSCC cases attributed to them. In stark contrast, HPV-associated HNSCC (HPV+ HNSCC) cases have been steadily increasing, with ~80–90% of oropharyngeal squamous cell carcinomas (OPSCCs) being HPV+ [11, 12]. HPV negative HNSCC (HPV- HNSCC) tumors frequently harbor alterations in *TP53* and *CDKN2A*, although several other tumor suppressor genes are also commonly mutated (*FAT1*, *NOTCH1*, *KMT2D*, *NSD1*, and *TGFBR2*) [2, 13]. Notably, *RAS* alterations are very rare in HPV− HNSCC despite carcinogen exposure, and *PIK3CA* is the only frequently mutated oncogene [13].

While the rates of mutation appear to be similar between HPV+ HNSCC and HPV− HNSCC [14], distinct mutational patterns exist between the two entities. Specifically, HPV− HNSCC tumors often present with *PIK3CA*, *TP53*, and *CDKN2A* alterations [13], while HPV+ HNSCC tumors have been shown to exhibit *PIK3CA*, *TRAF3*, *CYLD,* and *E2F1* alterations [2, 15]. Several recent advances in our understanding of the role that HPV infection plays in OPSCC tumorigenesis have changed the field dramatically [16]. HPV16 is the most prevalent strain among HPV+ HNSCC malignancies [17], accounting for ~90% of all HPV+ HNSCC cases. Akin to cervical squamous cell carcinoma in women [18], E6 and E7 are the key oncogenes driving HPV+ oropharyngeal tumor development [19] by inactivating p53 and pRb, respectively [20], and their ability to subsequently induce proliferation and malignant transformation of epithelial cells [21, 22]. Notably, the molecular pathways driving HPV+ HNSCC tumors are also associated with distinct clinicopathological characteristics such as age at diagnosis, immunologic profile, and microbiome status [23, 24]. Thus, patients with HPV+ and HPV− HNSCCs require different treatment strategies in the management of disease [25, 26]. Moreover, the development of HPV vaccines has raised the hopes for more effective prevention and management of HPV-associated neoplasms, including those originating within the head and neck epithelia [20]. However, until preventative vaccination is broadly adopted within the general population, the incidence of HPV+ OPSCCs is expected to continue to rise [27].

While the survival for HNSCC patients has improved over the past decades, it remains one of the most lethal malignancies worldwide, with 444,339 reported deaths in 2020 [2, 3]. Early-stage HNSCC is usually managed surgically, and results are optimal in this group with long-term survival rates exceeding 80% in some cohorts [2, 28, 29]. In laryngeal carcinoma in particular, primary radiotherapy is also an option [2, 29, 30]. However, pathologic risk features (e.g., extranodal extension and perineural invasion) determine the need for adjuvant treatment (radiotherapy and/or cisplatin-based chemotherapy). In contrast, late-stage disease and recurrent/metastatic disease remain difficult to manage despite an expansion in available treatment options over the past two decades, and patient prognosis is generally dismal [2, 29].

Targeted therapy [31] and immunotherapy [32, 33] have proven effective for only a small subset of the patient population in prolonging survival. Efforts to unravel the molecular pathogenesis of HNSCC through The Cancer Genome Atlas Project (TCGA) and other large scale genomic and transcriptomic analyses have identified a significant number of potentially actionable targets (e.g., PI3K, NOTCH1, TRAF3), although there remains a dearth of effective therapies [19, 34]. Thus, the current treatment landscape underscores the need for tools that can reliably recapitulate the molecular and cellular complexities of HNSCCs. These include unique genetic and transcriptional differences, as well as molecular and cellular heterogeneity between HNSCC subtypes, which all contribute to a wide diversity in treatment responses that need to be accounted for in order to enable the development and preclinical validation of new therapeutic options.

Unlike HNSCCs, SGCs only account for <0.5% of all cancers and around 3–5% of all head and neck cancers [35]. They arise in any of the three major salivary glands (submandibular, sublingual, and parotid gland) or the minor glands, with mucoepidermoid carcinomas (MEC), adenoid cystic carcinomas (AdCC), and polymorphous adenocarcinomas being the most frequent histopathological subtypes [36, 37]. Notably, SGCs display remarkable histologic heterogeneity that presents significant challenges in their diagnosis and management [38]. Surgical resection and adjuvant radiotherapy remain the primary treatment modality, however, there is a lack of systemic treatment options largely in the recurrent or metastatic disease setting [39]. The rarity of SGCs and their vast clinicopathological diversity impedes large-scale patient accrual to conduct prospective trials. Furthermore, the published randomized clinical trials have amalgamated different histological types and hence the performance of therapy can be limited. Consequently, there is an unmet need of subtype-specific approaches for the management of SGCs. Thus, in vivo models that can recapitulate salivary gland carcinogenesis are indispensable in identifying the cellular and molecular mechanisms to develop tumor subtype-specific treatments.

TC comprised 2.3% of all new cancer diagnoses in 2022 and accounted for 0.4% of all cancer deaths [40]. Specifically, in HNC, thyroid malignancies accounted for roughly 41% of all diagnoses in 2020 (*n* = 586,202 worldwide), but only 9% (*n* = 43,646) of head and neck cancer-associated deaths [41]. Fortunately, TC mortality remains low, with 5-year survival rates of >98%, although certain subtypes of TC are classified among the deadliest malignancies described in medicine. The thyroid gland is composed of several cell types, each with different histology. Follicular and epithelial cells are the major cell types that function to concentrate iodine and produce thyroid hormones. C-cells or para follicular cells are neuroendocrine cells that synthesize calcitonin. Molecular subtyping has aided in the classification of follicular cell-derived neoplasms including differentiated TC: papillary thyroid carcinomas (PTC) and follicular thyroid carcinomas (FTC), poorly differentiated thyroid carcinoma (PDTC), and undifferentiated anaplastic thyroid carcinoma (ATC). PTC is the most common subtype of TC accounting for about 80% of all TC cases, followed by FTC (about 15%), PDTC (>5%) and ATC (1–2%) [42]. Differentiated TC in general has excellent prognosis as opposed to the more aggressive PDTC and ATC that account for half of the TC-related deaths [43]. Medullary thyroid carcinoma (MTC) is a rare type of TC representing about 4% of all TC that is derived from C-cells [44]. About 20–25% of MTCs can arise due to an inherited syndrome, such as Multiple Endocrine Neoplasia (MEN) type 2A or type 2B or familial MTC [45, 46]. Management of TC differs according to the type of cancer. Surgery, with or without radioiodine therapy, is the primary treatment option for differentiated TC. While MTC is usually treated with upfront surgery, systemic targeted therapy can be given in the locoregionally advanced stages, either in an adjuvant or neoadjuvant setting [47]. ATC is commonly unresectable and metastatic at the time of diagnosis, making systemic treatment, with or without external beam radiation therapy, the first-line therapy. Development of targeted therapies for progressive metastatic radioactive iodine refractory differentiated TC, metastatic MTC, and ATC patients resulted in improved clinical outcomes and survival [48–50]. Comprehensively evaluating such therapies for advanced and aggressive TC has been historically challenging in clinical trials due to the rarity of ATC and MTC. Having reliable and established in vivo models for TC can help expedite novel therapeutic development.

Given the challenges associated with treating patients with any of the above HNCs, the identification, selection, and/or development of in vivo models suitable for interrogating the unique pathobiology of these cancers is crucial. Compared to other animal models, the development of mouse models offers easier genetic engineering, higher rates of successful tumor formation, faster reproduction, and lower development and maintenance costs [51]. Genetically engineered mouse models (GEMMs) in particular, can recapitulate most of the heterogeneous characteristics of various tumors including the tumor microenvironment [52, 53]. In head and neck cancer, progress in the development of GEMMs has been slower than in other major cancer types, such as melanoma [54], non-small cell lung carcinoma [55], and pancreatic adenocarcinoma [56]. This is due in part because HNCs are primarily characterized by genetic instability leading to chromosomal translocations or the frequent loss or gain of chromosomal regions involving tumor suppressors (e.g., *CKND2A*, *TP53*) but many available GEMMs employ oncogene overexpression instead (e.g., *RAS*) to model HNC carcinogenesis [2]. However, several GEMMs with or without concurrent carcinogen exposure are now available, enabling research questions regarding many aspects of carcinogenesis [57, 58], treatment response and resistance [59–61], and relapse/recurrent disease [62].

GEMMs enable researchers to study the biological effects of oncogene overexpression or tumor-suppressor gene inactivation on downstream signaling and target genes in a defined genetic background [63]. Thus, tumors that develop in GEMMs are more likely to be histologically and genetically accurate representations of human cancer. Constitutive knockout transgenic mice were developed by depleting or silencing the gene of interest in germline cells leading to permanent inactivation of the target gene in every cell of the organism. However, this is inconsistent with the HNSCC mutational landscape, and the timing of gene loss is critical in carcinogenesis. To overcome this limitation, conditional and conditionally inducible models were developed that enable tissue- and time-specific regulation of the gene of interest by external stimulation by chemicals or virus [64]. Exposure to the carcinogen 4-NQO has been integrated with certain models for development of spontaneous tumors. DNA damage by chemicals occurs randomly, thus the stochastic nature of 4-NQO induced mutations makes these GEMMs less genetically defined [65]. It is therefore important to note that the approach employed in GEMM establishment and implementation, recapitulates only certain aspects of the disease course with accuracy. Moreover, models are usually developed for specific tumor sites within the head and neck region (e.g., oral cavity). Thus, appropriate model selection is essential for addressing specific research questions. At the same time, as more and more information on the molecular background of HNCs comes to light, new models which accurately represent the underlying mechanisms of head and neck carcinogenesis are necessary to improve our understanding of the disease and develop improved precision medicine therapies. In this review, we present a critical overview of the existing head and neck cancer GEMMs and discuss strengths and limitations of each model with respect to the research questions under investigation and the different anatomic sites where these cancers can develop.

## Head and neck squamous cell carcinoma

### Oral cavity

**Fig. 1:**
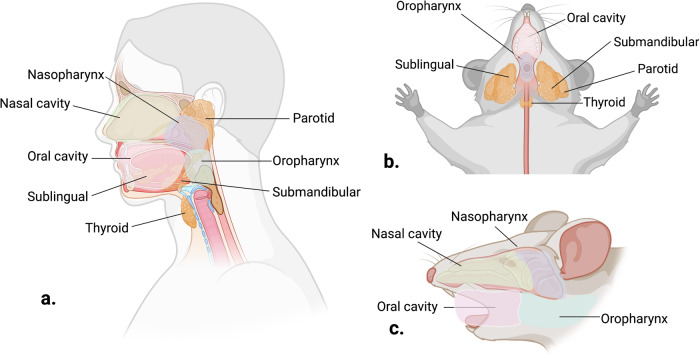
Schematic representation of the head and neck anatomy. **a** Sagittal view of the human head and neck anatomy. **b** Ventral and **c** Lateral view of mouse head and neck anatomy. Created with BioRender.com.

**Fig. 2:**
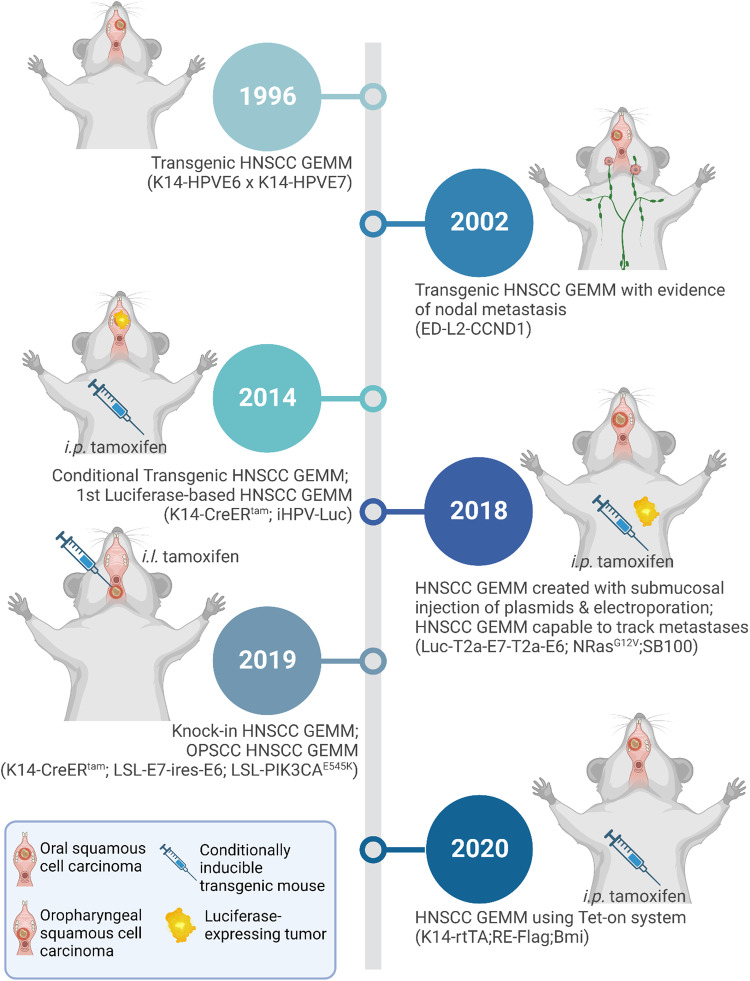
Timeline of genetically engineered mouse models (GEMMs) of head and neck squamous cell carcinoma (HNSCC). Created with BioRender.com.

The majority of currently available HNSCC GEMMs are intended for modeling oral SCC, with some of them developing tumors exclusively in the tongue and others developing tumors both in the tongue and the buccal region [58, 66–81] (Fig. 1 and Table 1). Transgenic models were the first to be developed, followed by several more sophisticated conditional and inducible models in recent years and are summarized accordingly below (Fig. 2).

**Table 1:** Characteristics of the genetically engineered mouse models for head and neck squamous cell carcinoma.

Approach	Mouse strain	Genotype	Phenotype	Tumor location	Latency and Penetrance	Advantage	Limitation	Ref.
Submucosally injection of plasmids followed by electroporation	C57BL/6NCr, FVB/NCr, BALB/cAnNCr, Nude mice	Luc-T2a-E7-T2a-E6; NRas^G12V^; SB100	Spontaneous HPV buccal tumors in immunodeficient mice, and immunocompetent mice subjected to early immune suppression.	Oral cavity	17 days ~100%	Time-saving without breeding. Capable to track metastasis. Capable to screen via SB system Determine the therapeutic effects of DNA vaccine	CD3 depletion is required. Lymph node metastases only detect in nude mice. Tumor morphology more similar to sarcomas than SCCs Ras is rarely mutated in human HPV+ HNSCC	[66]
Transgenic mice	FVB/N	K14::HPV16E6; K14::HPV16E7.	Wrinkled skin, loss of hair, epithelial hyperkeratosis and hyperplasia, and skin tumor.	Oral cavity	6–12 months ~10% for E6 or E7 solo ~40% for E6 + E7 Combo	E6 + E7 transgenic model is far more efficient to induce tumorigenesis compared to E6 or E7 transgene alone. E6/E7 mice treated with low dose 4NQO developed esophagus or tongue tumors similar to HPV positive human HNSCC.	Transgene activation in embryo induces developmental defects and immune tolerance. Phenotypes do not recapitulate HPV+ HNSCC in adult humans In general the incidence rate of skin tumors is very low, majority are hyperplasia.	[67–72]
Conditional transgenic mice	FVB/NJ	K14::CreER^tam^; iHPV-Luc (CAG::LSL-E6/E7-ires-Luc); CAG::LSL-Kras^G12D^	Oral tumors in KHR mice	Oral cavity	10–15 days ~100%	Co-expression of luciferase and oncogene E6/E7, thus capable of monitoring tumor growth real time. First research measuring effects of Rapamycin in HNSCC growth.	E7 expression dependent on alternative splicing of the E6 transcript E7 expression is not validated Tamoxifen is intraperitoneally injected, leading to systemic activation of Cre. Ras mutation is rare in HPV+ HNSCC.	[73]
Transgenic and knockout mice	C57BL/6	L2D1 (ED-L2::CCND1); TP53^+/-^ or TP53^-/-^	Squamous cell cancers in mouth (buccal mucosa), tongue, upper esophagus, or lower esophagus	Oral cavity	3–12 months 61% for L2D1^+^;p53^+/–^ mice	25% of cancers had evidence of metastasis to lymph nodes in L2D1^+^/p53^+/–^ mice	Lower frequency of buccal SCC compared to tongue SCC	[74]
Conditionally inducible transgenic mice	C57BL/6	K14-rtTA; TRE::Flag-Bmi-1	Bmi1 + 4NQO mice exhibited higher grade and larger number of oral tongue lesions	Tongue	~25 weeks. ~100% for Bmi1 + 4NQO mice.	First OSCC model using Tet-on system	Bmi1 expression without 4NQO was incapable of forming tumors Bmi1 amplification or overexpression is rare in HSNCC samples (6% in TCGA)	[78]
Conditionally inducible transgenic mice	CBA/Ca	K14::CreER^TM^; K5::LSL-rtTA; Tet::E6/E7; Rosa26::LSL-PIK3CA^H1047R^	mild or moderate dysplasia, severe dysplasia and OSCC	Tongue	9–20 weeks 60% for PIK3CA + 4NQO mice.	PIK3CA + 4NQO model develops cancer faster and exhibits the most consistent lymphocytic infiltration, compared to 4NQO and PIK3CA + E6/E7 model.	PIK3CA-E6/E7 model formed dysplasias but not OSCC tumors Tamoxifen is not as effective as 4-OHT Overdose treatment of Doxycycline CreER^TM^ and rtTA target to different epithelial populations (K14 vs. K5)	[58]
MmuPV1 infection, UVB radiation, 4NQO.	FVB/N, Nude mice, NSG mice	MmuPV1+	Invasive SCC after concurrent exposure to UVB and 4NQO	Tongue	~6 months 25% in MmuPV1 + 4NQO nude mice. 12.5%–43% in MmuPV1 + UV + 4NQO FVB/N mice	First HNSCC model based on virus infection, capable for short-term mass production.	Low incidence rate of invasive SCC with MmuPV1+UVB + 4NQO. Most assays are performed in immunocompromised mice. UVB radiation is not clinically relevant to HNSCC	[80]
Conditionally inducible transgenic mice	C57BL/6	K5.GLp65; Tata.PIK3CA;	Oral tumors, lymph node and/or lung metastasis	Oral cavity	5–6 months for PIK3CA + 4NQO mice (39%), and 100% at 12 months.	First PIK3CA overexpression model in HNSCC, and PIK3CA overexpression is inducible and within physiological range.	Overexpression of PIK3CA alone is not sufficient to initiate HNSCC formation	[87]
Conditionally inducible transgenic mice	C57BL/6	K5.GLp65; Tata.PIK3CA;	Oral tumors, lymph node and/or lung metastasis	Oral cavity	5–6 months for PIK3CA + 4NQO mice (39%), and 100% at 12 months.	First PIK3CA overexpression model in HNSCC, and PIK3CA overexpression is inducible and within physiological range.	Overexpression of PIK3CA alone is not sufficient to initiate HNSCC formation	[81]
Conditionally Knock-in	C57BL/6J	Rosa26::LSL-E7-ires-E6; Rosa26::LSL-PIK3CA^E545K^; KRT14-Cre^tam^	Spontaneous HPV oropharyngeal tumors in immunocompetent mice	Oropharynx	6–8 weeks for E6/E7;PIK3CA^E545K^ mice, 10-20 weeks for E6E7 + 4NQO mice	First knock-in mouse model of OPSCC. Balanced E6 and E7 expression. Preneoplastic lesion development with spontaneous conversion to cancer. First HPV+ HNSCC model to involve PIK3CA^E545K^ mutation.	Oropharyngeal SCCs were often carcinomas in situ although invasive cancer with regional lymph node metastasis does occur	[79]

Overexpression of Cyclin D1 (*CCND1*) has been reported as an early event in oral and esophageal SCC [82, 83]. An Epstein-Barr virus (EBV) lytic promoter ED-L2 resides within the 3′ UTR of the EBV latent membrane protein-1 gene and extensive characterization of its promoter activity revealed tropism for human aerodigestive epithelial cells including the tongue, esophagus, and forestomach. Thus, the ED-L2 promoter-mediated expression of *CCND1* combined with *Trp53* knockout leads to the development of SCC within the buccal mucosa, tongue, and esophagus of C57BL/6 mice [84]. However, buccal SCC develops far less frequently than tongue SCC. Notably, this model was also able to produce nodal metastasis, with approximately a quarter of mice displaying positive lymph nodes. A major caveat of this model is that while *CCND1* is reported to be overexpressed in HNSCC [85, 86], it remains unclear if it is a main driver of carcinogenesis at these sites [34]. Thus, while its combination with *Trp53* loss in mice is able to produce squamous carcinomas within the oral cavity, the molecular landscape of these tumors in humans differs substantially from those commonly associated with HPV– HNSCC.

GEMMs have successfully used combinations of oncogene overexpression and carcinogen exposure to promote malignant tumor development. Specifically, Kalish et al. [78] created a conditional and doxycycline-inducible C57BL/6 model where Bmi1 is overexpressed in basal epithelial cells and that forms tongue SCC upon concurrent 4-Nitroquinoline 1-oxide (4NQO) exposure. While the conditional nature of this model limits transgene impact to squamous epithelia, administration of doxycycline to the drinking water leads to transgene activation not only in the mucosal epithelia of the oral cavity but also in the cutaneous epithelia of the skin. Perhaps more importantly, *BMI1* overexpression is only reported in <4% of HNSCC patients in the TCGA and is therefore not considered a primary driver of HNSCC oncogenesis [34]. This is further highlighted by the inability of Bmi1 to form malignant tumors without concurrent 4NQO exposure. Importantly, some alterations induced by 4NQO exposure in mice such as Hras mutations are mutually exclusive with BMI1 overexpression in human HNSCCs, further limiting the utility of this GEMM. *PIK3CA* mutations and/or overepression are commonly identified in >40% of all HNSCCs in the TCGA and this pathway is associated with cell survival and malignant transformation. Du et al. developed a conditionally inducible GEMM where *PIK3CA* overexpression is regulated by the anti-progesterone and anti-glucocorticosteroid agent RU486 (mifepristone). While in this model PI3K pathway deregulation alone is insufficient for tumor development [81], concurrent RU486-induced PIK3CA overexpression and 4NQO administration results in over 40% of tumors exhibiting increased invasion and metastasis. This model of concurrent carcinogen exposure and PIK3CA overepression closely recapitulates drivers of human HPV– HNSCC, where tobacco exposure is a common carcinogen and of PI3K overexpression and/or hyperactivation of its downstream target PDK1 promote a more aggressive biological behavior [87].

### Oropharynx

Numerous GEMMs intended to model HPV+ HNSCC have been developed by employing the HPV oncogenic proteins E6 and/or E7 [67–73, 84]. While initially developed as an HPV16 model for cervical SCC, these animals were also used to model HPV+ HNSCC upon identification of an etiologic role for HPV in oropharyngeal HNSCCs. A keratin 14 promoter (K14) was used to express the viral oncogenes in FVB/N mice, where the independent expression of both E6 and E7 in bi-transgenic mice (i.e., K14-E6; K14-E7) demonstrates greater transforming potential than each oncogene alone. However, the constitutive expression of these oncogenes only results in a hyperkeratotic and hyperplastic skin phenotype, with wrinkled and hairless skin. Also, embryonal transgene activation induces developmental defects and immune tolerance. An improved HPV+ HNSCC GEMM based on combination of inducible expression of HPV E6/E7 and oncogenic *Kras*^*G12D*^ (i.e., LSL-Kras^G12D^) in FVB/NJ mice was developed by Zhong et al. [73]. In this model, E6/E7 expression is conditionally induced in basal epithelial cells using K14-CreER and intraperitoneal tamoxifen injection. Interestingly, this model incorporates an internal ribosomal entry site (IRES) with luciferase (i.e., *CAG::LSL-E6/E7-IRES-Luc*) which enables E6/7 expression and the subsequent tumor growth to be visualized in real time in vivo. However, while KRAS is a powerful oncogene, KRAS mutations do not occur in HNSCC and HRAS mutations are only found in a small percentage of HPV– HNSCCs, and not in HPV+ cases. Moreover, while this model attempts to produce E6 and E7 overexpression, E7 expression relies on the alternative splicing of E6 (E6 I*) and expression of this transcript or E7 protein levels are not validated. Tan et al. developed another HPV + GEMM that incorporates inducible *PIK3CA* (LSL-PIK3CA^H1047R^) with concurrent E6/E7 oncogene expression and 4NQO exposure to form oral tongue lesions [58]. Notably, this GEMM uses a *PIK3CA*^*H1047R*^ transgenic allele but this mutation is not common in HPV+ HNSCCs.

Unconventional GEMMs have also been developed that utilize the Sleeping Beauty transposon mutagenesis system (SB100) which provides an opportunity to screen for cooperating oncogenes/tumor suppressors. For example, the Luc-HPVE6/E7-NRAS^G12V^-SB100 model is a constitutive HPV16 E6/E7 model that employs luciferase to track cells in the oral cavity and draining lymph nodes that get transfected with the SB100 plasmid system [66]. This model uses submucosal delivery via injection, thus avoiding a more time-consuming breeding approach. However, the model depends on mutant NRAS to form tumors. As The Cancer Genome Atlas (TCGA) and others have reported, RAS is infrequently involved in HNSCC [2, 34], thus limiting the relevance of this model of human HNSCC. Acknowledging this limitation, Lin et al. replaced NRAS^G12V^ with constitutively active AKT, a PI3K downstream target but tumor formation is only reported among immune depleted mice [66]. Of note, NRAS mutant tumors only develop after depletion of CD3 + T-cells with a sarcoma-like histology consisting of spindle-shaped tumor cells instead of squamous cells. Development of lymph node metastases and the non-invasive visualization offered by the luciferase reporter are important features of this model.

While the aforementioned models have employed HPV oncogenes, these GEMMs have only been shown to develop oral cavity tumors and high-risk HPV infections have been associated almost exclusively with oropharyngeal SCC (OPSCC) in humans. In stark contrast, Carper et al. reported a conditional and inducible knock-in model using C57BL/6J mice, that offers balanced post-natal E6 and E7 expression with the appearance of spontaneous oropharyngeal tumors (Table 1). This was achieved by establishing a protocol for local administration of tamoxifen to the submucosa of the murine equivalent of a human oropharyngeal region to achieve mosaic transgene expression [79]. While most tumors were early-stage carcinomas, frank malignancy is observed when the mice are exposed to chemical carcinogens (e.g., 4NQO) or by combining E6/E7 overexpression with expression of mutant *PIK3CA*, specifically *PIK3CA*^*E545k*^. Consequently, this is the first model to use a knock-in system to accurately recapitulate many of the unique molecular features of HPV-associated tumors of the oropharynx [79]. Additionally, robust leukocyte infiltration was observed in response to exogenous viral antigen exposure in pre-malignant lesions and the tumors exhibited histopathological and molecular features observed in human HPV(+) OPSCC [79]. Thus, this model forms the full spectrum of premalignant lesions (mild to severe dysplasia) and OPSCC marked by consistent lymphocytic infiltration, making it ideal for studies of the tumor immune microenvironment and host-tumor interactions.

### Nasopharynx

Nasopharyngeal carcinomas (NPCs) have been stratified into three subtypes: keratinizing SCC, non-keratinizing SCC, and undifferentiated or poorly differentiated carcinoma. The etiology of non-keratinizing NPC is associated with EBV infection [88]. QingLing et al. established L2/LMP1^B95 – 8^/EGFP transgenic mice using the EBV ED-L2 promoter to drive expression of the EBV Latent membrane protein 1 (LMP1) in aerodigestive epithelial cells. LMP1 expression inhibits Wilms’ tumor gene on the X chromosome expression, leading to increased expression of β-catenin in these mice. Squamous epithelial hyperplasia and atypia was observed in the nasopharynx and oropharynx of founder (F0) and first generation (F1) animals at 50% penetrance; however, progression to neoplasms was not observed [89]. Therefore, the development of GEMMs to model human NPCs is highly warranted.

### Current limitations of HNSCC GEMMs

Despite technological improvements in surgery [90] and radiation [91, 92] and the rapid development of immunotherapies and targeted therapies in recent decades [2, 29, 31], clinical outcomes of HNSCC patients remain mostly unchanged, especially in HPV− cases where tumor mutational burden is higher [2, 3]. On the one hand, tumors in the oral cavity, oropharynx, and larynx have different clinical features and biological characteristics. On the other hand, HPV+ and HPV− HNSCC are now considered distinct disease entities with different genetic landscapes, biologic behaviors, treatments, and prognoses [21]. This is also reflected in the most recent 8th Edition of the TNM classification system of the American Joint Commission on Cancer [93]. Therefore, personalized treatment based on HPV status, tumor histology, and the specific genetic alterations of each tumor will improve the survival and life quality of HNSCC patients.

While the intrinsic complexity of HNSCC tumor cells is the key determinant driving tumorigenesis, the role of the tumor microenvironment and tumor immune microenvironment is more and more acknowledged as a critical parameter of tumor progression [94, 95], treatment response [96] and prognosis [97]. In GEMMs, normal cells undergo the multi-step process of malignant transformation and thus allow for the development of a stromal response that can recapitulate the complex tumor microenvironment and immune microenvironment. This enables GEMMs to accurately recapitulate tumor growth, treatment responses, and tumor-stroma and tumor-immune system interactions, features often lacking in xenograft or syngeneic transplant models [53]. However, while currently available GEMMs provide invaluable information on head and neck tumorigenesis and play critical roles in identifying and testing novel therapeutic approaches, most HNSCC GEMMs suffer from low tumor incidence. This largely hampers the reproducibility of preclinical results in clinical follow-up studies. Improving current models to generate high-efficiency, versatile HNSCC GEMMs is essential to understand the molecular mechanisms and develop new therapies. To achieve this, there are several obstacles that must be overcome.

E6/E7 sequences from HPV are widely used to generate transgenic mice prone to HNSCC tumorigenesis, aiming to recapitulate human HPV+ HNSCC. However, the random integration into the mouse genome, often as concatemers and/or at multiple loci, may lead to genetic instability or even phenotypic instability during breeding, as has been observed in studies where E6 transgenic mice were crossbred with E7 transgenic mice [73]. In comparison, the conditional knock-in of E6/E7 cDNA expression cassettes into the Rosa26 locus induced stable and balanced E6 and E7 expression [79]. Even so, E6 and E7 expression could only induce hyperplasia in most cases. Malignant transformation is rarely achieved without additional driver mutations or exogenous carcinogens, indicating that E6 and E7 oncoproteins are incapable to initiate and promote HNSCC tumorigenesis without further triggers. In this regard, a model that would include the full-length HPV genome could potentially more accurately recapitulate the pathogenesis of HPV+ HNSCC and induce tumors without the need for concurrent exposure to carcinogens (e.g., 4NQO).

The TCGA dataset includes 504 HNSCC samples with integrated mutation and copy number alteration (CNA) data. Notably, TP53 is mutant in 72% of samples, CDKN2A is altered in 54% of samples (22% mutation and 32% deletion), and PIK3CA is altered in 39% of samples (18% mutation and 21% amplification). Moreover, alterations of these 3 genes cooccur, indicating they may all be essential for HPV− HNSCC tumorigenesis. GEMMs harboring alterations of Tp53, Cdkn2a, and Pik3ca are available, *K14-Cre*^*ERT2*^*; R26-LSL-Pik3ca*^*H1047R*^*; Trp53*^*fl/fl*^*; Cdkn2a*^*fl/fl*^ mice could be generated to study the combinatorial effects of these three drivers. Considering that developing this strain requires several generations of breeding and thus is very time and labor consuming, CRISPR knockouts could be an alternative approach. Specifically, traditional embryonic stem cell (ESC) mediated targeting strategies employ homologous DNA recombination to perform genetic recombination followed by injection of these modified ESCs into blastocysts to generate chimeric embryos within pseudopregnant mice [98]. This process is highly technical and time-consuming since these chimeric animals must then be mated with wild-type mice to screen for passage of the genetic information to subsequent generations which can take 10 months to over 1 year. In contrast, CRSIPR/Cas9 methodologies employ a guide RNA (gRNA) mediated targeting strategy and a homology-directed repair mechanism to repair the damaged double-stranded DNA which can facilitate either knockin or deletion of genetic material. Specifically, Cas9 transgenic animals [99] can be crossed with an existing HNC GEMM and these compound mutant mice can then be used to study several knockout mutations following delivery of gRNA(s) targeting the gene of interest. Importantly, the desired genetic manipulation is achieved within the first generation of offspring reducing breeding schema and time needed to generate models to less than 4 months. Using this CRIPSR-based approach, other potential driver genes including CCND1 (25% amplification), SOX2 (16% amplification), NOTCH1 (18% mutation and 4% deletion), and FAT1 (23% mutation and 8% deletion) can be examined in less time. Interestingly, PIK3CA and SOX2 are frequently co-amplified since they are both localized on chromosome 3q26, but whether overexpression of PIK3CA and SOX2 have synergistic effects in HNSCC is unknown. To determine the roles of these frequently amplified genes in vivo, CRISPR activation (CRISPRa) could be an effective approach.

The availability of the Cre-LoxP (for instance, *K14-Cre*^*ERT2*^) and Tet-on systems enabled the development of HNSCC GEMMs where tumor development is induced in a spatiotemporal manner. However, the exact site and timing for HNSCC initiation could be further optimized. First, most HNSCC models activate Cre 4–8 weeks after birth, which may be too early given that HNSCC is a disease predominantly affecting the elderly. Second, to increase the effectiveness and specificity of Cre activation, intraperitoneal injection of Tamoxifen could be replaced by topical administration of 4-Hydroxytamoxifen (4OHT), including brushing in the oral cavity or dripping in the pharynx, or by targeted submucosal delivery of Tamoxifen as performed by Carper et al. [79]. Furthermore, adenoviral or lentiviral delivery of Cre to the oral cavity or pharynx could be used to initiate genetic recombination of driver alleles in squamous cells, which would decrease genotype complexity of experimental animals and thus shorten the breeding process.

Current mouse models indicate that HPV infection alone may not be capable of initiating HNSCC and additional exogenous stimuli are essential for malignant transformation [58, 78, 81, 87]. 4NQO, a quinolone derivative to induce DNA lesions, has been used in some HNSCC models as a carcinogen to enhance HPV-induced tumorigenesis (Table 1). However, the use of 4NQO in HPV+ models has some shortcomings. First, 4NQO is a substitute for tobacco exposure, which is not considered a major risk factor for the majority of HPV+ HNSCC where incidence rates appear to be increasing primarily in male non-smokers. Second, transcriptional or epigenetic deregulation rather than genetic mutation alone may drive HPV+ HNSCC carcinogenesis. Thus, a comprehensive screening approach using siRNA or cDNA libraries may help to better understand HPV+ HNSCC initiation. The roles and mechanisms for many other natural carcinogens closely related to HPV− HNSCC, including PAH and nitrosamine in cigarettes, tannins in areca nut, as well as alcohol, are yet to be determined in vivo. These carcinogenic substances could be used to recapitulate the exposures of HPV− HNSCC to develop models that provide more accurate information on various questions related to HNSCC prevention and treatment. Third, most of the models failed to recapitulate the molecular and histological features of human HNSCC (Table 1), with the notable exception of Bmi-1 conditionally inducible transgenic mice, exposed to 4NQO [78].

## Salivary gland carcinoma

Despite the remarkable heterogeneity of SGC, there have been several attempts to recapitulate these tumors in vivo. Conventionally, the mouse mammary tumor virus long terminal repeat promoter was used to develop GEMMs of breast cancer. Since this promoter directs gene expression to secretory glands, extra-mammary gland tumors were reported in the salivary glands of these mice [100]. Consequently, new murine models of salivary gland tumorigenesis driven by the MMTV promoter were characterized in detail (Table 2). Brodie et al. developed a MMTV-driven c-neu transgenic mouse strain that exhibits increased rates of parotid tumor onset on a Trp53-deficient background [101]. Moreover, Zboray et al. employed MMTV-*rtTA* animals crossed to TetO-*Akt3* transgenic mice to direct Akt3 overexpression to the salivary glands. These bi-transgenic mice (MMTV-*tTA*; TetO-*Akt3*) exhibit tumors upon doxycycline administration that were characterized as AdCC [102]. Notably, incidence of AdCC in the salivary glands is frequently associated with recurrent translocation and oncongenic fusion of v-myb avian myeloblastosis viral oncogene homolog (MYB) and nuclear factor I/B (NFIB) and/or overexpression of the MYB or MYBL1 transcripts [103–105]. Hence, to examine AdCC, a MYB-NFIB fusion construct was conditionally expressed in the salivary gland by crossing to MMTV-Cre mice. There is no robust expression of MYB-NFIB transcripts in salivary gland tissues nor is malignancy observed [106]. The introduction of a conditional Trp53 knockout allele also fails to yield salivary tumors in these animals even though MYB-NFIB; MMTV-Cre; p53^+/fl^ mice present with tumors in the upper mammary glands [106]. A MYB-NFIB; MMTV-Cre; Cdkn2a^+/−^ GEMM also does not develop salivary tumors and the predominant phenotype of this model is B-cell leukemia [107]. Even though MYB-NFIB fusions have been reported to be a key event in the development of AdCC in humans, these studies failed to recapitulate its role in inducing SGCs in mice. More robust attempts to target MYB-NFIB to salivary gland-specific cell types and/or addition of concurrent mutations in genes such as NOTCH1, CDKN2B, NRAS, HRAS, ALK, c-KIT, TSC, PIK3CA, PTEN, or NF1 might be required to promote AdCC initiation and progression [108].

**Table 2:** Characteristics of the genetically engineered mouse models for salivary gland carcinomas.

Approach	Mouse strain	Genotype	Phenotype	Tumor location	Latency ; Penetrance	Advantage	Limitation	Ref.
Conditional transgenic mice	FVB/N	MMTV‐tTA/TetO‐Akt3	AdCC	Salivary glands(sublingual, submandibular, and parotid glands)	8–12 weeks; ~100%	Assess the therapeutic potential of small molecule inhibitors against proto-oncogene Akt3-driven AdCC	MMTV promoter targets genes predominantly in mammary glands rather than salivary glands.	[102]
Conditionally inducible transgenic knockout mice	C57BL/6	K5CrePR1 PtenSmad4^SG-KO^ Pten^SG-KO^ or Smad4^SG-KO^	AdCC SDC, SAdSCC PA	Salivary glands NOS	8–10 months; ~100% ~70%; ~42%	Improved penetrance of developing SGTs. Application of RU486 directly into salivary glands avoids the generation of tumors in other organs. Effect of mTOR and TGFβ pathway inhibition can be tested.	The same combination of gene knockout caused the development of three distinct histopathological forms of tumors	[109]
Transgenic mice	FVB	MMTV-LTR-Cre+/−/PLAG1+/−	PA	Salivary glands (Submandibular gland)	5 weeks; ~100%	Tumor progression can easily be followed in the time.	PLAG1 activation starts during embryonic development unlike in humans where clonal activation of PLAG1 expression occurs long after birth.	[156]
Transgenic mice	FVB (strain TG.NK)	MMTV-c-neu; p53^+/-^	NOS	Salivary glands (Parotid gland)	35 weeks; ~90%	Loss of p53 allele had a minimal effect on the rate of breast tumor formation in c-neu transgenic mice.	p53 deletion led to numerous spontaneous tumors in other sites.Histology of tumor not specified.	[101]
Conditional transgenic mouse	FVB FVB/SLJ B6/129	MMTV-Cre/Apc^+/-^/Pten^-/-^ or Apc^-/-^/Pten^-/-^	ACC	Salivary glands (Parotid gland)	6–25 weeks; ~100%	None of the mice, regardless of genotype, developed mammary gland tumors 100% penetrance and short latency	35% of mouse tumors contained areas of CK-6–positive squamous metaplasia which is not the case in human ACC.	[116]
Conditional transgenic mouse	FVB/J	MMTV-Cre; CRTC1-MAML2 (mCre-CM(+))	MEC	Salivary glands NOS	3–9 months; ~100%	First GEMM for fusion-positive human MEC	Focal nature of tumors and tumor latency	[115]
Transgenic mice	CB6F1/J	Smgb-Tag	ICD Adenocarcinoma	Salivar glands (Submandibular Gland)	3–12 months; ~50%	Morphologic tracking of the premalignant process Provides direct evidence implicating specific cell types in the genesis of salivary tumors	The histological features of the mice adenocarcinoma resemble more to human acinic cell carcinoma Female Smgb-Tag mice developed tumors infrequently	[114] [113]
Double mutant mice	C57BL/6	K14-cre-β-cat^GOF^-Bmpr1a^LOF^	SCC	Salivary glands NOS	Rapid tumor development; Lifespan between postnatal day P75 and P90	Characterization of tumor-specific cells using single-cell transcriptomics. Being a Wnt-dependent model provides a genetically controlled setting to study mechanisms of tumorigenesis in vivo.	The histological subtype of the tumors that were generated is not specified.	[157] [110]
Chemically induced recessive point mutation	C3HeB/FeJ or *Justy* mutant mice	Homozygous for a point mutation in Gon4l gene	BCAC	Salivary glands NOS	≥6 months; ∼25%	Spontaneous intermediate-grade SGT formation at an incidence rate of 25%. Immunohistochemically similar to human SGTs composed of basal cells.	Lack of peripheral B-cells can likely contribute to tumor development. Relatively low penetrance of tumor formation and a long latency period.	[112]

Although numerous MMTV promoter-dependent models of SGCs have been developed, the appearance of mammary gland tumors has been the major limitation of these GEMMs. Therefore, more selective approaches that enable targeting of salivary gland cell types are highly warranted. Using an RU486-inducible keratin 5-driven Cre allele (K5-CrePR), the tumor suppressors Pten and Smad4 are specifically deleted in K5-positive basal epithelial cells in the salivary glands [109]. Deletion of either Pten or Smad4 results in spontaneous pleomorphic adenomas whereas deletion of both genes leads to the development of several subtypes of malignant SGCs with salivary AdCC being the most frequent. In addition, salivary duct carcinomas and salivary adenosquamous cell carcinomas are observed in the double knockout mice [109]. A K14-Cre-dependent β-catenin gain-of-function (β-cat^GOF^) and Bmpr1a loss-of-function (Bmpr1a^LOF^) double mutant model was developed using the β-catenin^lox(ex3)^ and Bmpr1A^flox^ alleles. While no tumors develop in the single mutant mice, double mutant mice develop aggressive squamous cell carcinomas (SCCs). These tumors form exclusively in the submandibular salivary glands requiring euthanasia after 75–90 days, and no tumors are observed in other K14 expressing tissues [110].

A mouse strain bearing a chemically induced recessive point mutation in the Gon4-like (Gon4l) gene was generated in which B lymphopoiesis is blocked at an early stage without hampering other hematopoietic cell development [111]. Mice homozygous for this mutation were named *Justy* (just T-cells) and in an independent study were shown to spontaneously develop SGCs with myoepithelial and basaloid differentiation. This model suggests an association between SGC development, downregulation of Gon4l mRNA transcripts and protein levels and/or the consequent depletion of B-cells, which can be further explored to understand cellular and molecular mechanisms [112]. The submandibular gland secretory protein b (Smgb)-SV40 T antigen (Tag) transgenic mouse model is another powerful tool for delineating molecular mechanisms underlying salivary gland tumorigenesis [113]. Using the neonatal Smgb promoter, a Tag transgene, which functionally inactivates p53 and Rb, is expressed in neonatal submandibular gland pro-acinar cells and terminal tubule cells and in the intercalated ducts of the adult gland. Tag expression in the intercalated ducts triggers progressive hyperplasia, dysplasia, and adenocarcinoma [114].

The importance of identifying oncogenic fusion genes for the development of treatment modalities is emphasized in a transgenic mouse model where Cre-regulated conditional expression of cyclic AMP-regulated transcriptional coactivator 1 and mastermind-like 2 fusion gene (CRTC1-MAML2) results in the formation of SGC resembling human MEC in histological and molecular features [115]. Even though this model is valuable to characterize MEC, it lacks the ability to target the expression of CRTC1-MAML2 to specific ductal cell populations since the Cre recombinase is driven by the MMTV promoter. A model displaying SGCs with remarkable morphological similarity to human acinic cell carcinoma was generated by conditional inactivation of the Apc and Pten tumor suppressor genes using MMTV-Cre, thereby constitutively activating the Wnt and PI3K/AKT/mTOR signaling pathways. Treatment with Rapamycin leads to the regression of the tumors in these mice, suggesting a role for mTOR in salivary gland acinar cell carcinoma development [116].

## Thyroid carcinoma

TC is the second most common HNC after HNSCC, and there is currently a broad array of treatment options that depend on TC type and individual clinicopathological characteristics [117]. A multitude of molecular and genetic analyses have revealed that thyroid tumorigenesis and progression involve multiple genetic alterations including point mutations of the BRAF or RAS genes and fusions of RET, NTRK, ALK PAX8, and PPARγ, ultimately leading to the activation of the MAPK and PI3K/AKT signaling pathways [118–120]. This understanding of TC genomics has supported the development of several TC GEMMs intended to model these genetic mutations and mimic the human condition, making these strains suitable for the preclinical evaluation of new therapeutic modalities for TC (Table 3). However, it is crucial to select an appropriate time-point during the disease process, at which the model should be tested for therapies against a specific histopathological subtype since de-differentiation has been observed in these models.

**Table 3:** Characteristics of the genetically engineered mouse models for thyroid carcinomas.

Approach	Mouse strain	Genotype	Phenotype	Latency; penetrance	Advantages	Limitations	Ref.
Knock-in of dominant negative thyroid hormone receptor gene	C57BL/6J	Trbeta^(PV/PV)^	PTC and ATC	50%; 6–12 months	Demonstrate pathological progression	Little clinical relevance to PTC/ATC	[121, 122]
Transgenic mice	FVB/N mice	Tg-BRAF2 and Tg-BRAF3	PTC PDTC	12–22 weeks; 93% (BRAF2); 25%–45% (BRAF3)	Useful to study dedifferentiation of PTC	Lack of distant metastasis. Elevated TSH levels.	[158]
Transgenic mice with doxycyline-inducible BRAFV^600E^ expression	FVB/N	Tg-rtTA/tetO-BRAF^V600E^	PCT	1 week after dox induction; 100%	Very short latency The timing of neoplastic induction can be controlled, thereby preventing juvenile tumor formation Malignancy manifests ubiquitously throughout the entire thyroid gland.	No nodal or distant metastases were observed suggesting insufficient oncogenic potential.	[124]
Transgenic mouse (Induced by Tamoxifen)	Mixed C57BL/6 and FVB/N	Thyro::CreER^T2^; BRaf^CA^	PTC	12 months	PTC display characteristic cytological features and protein marker expression of the cognate human disease	Stochastic BRAF^V600E^ expression due to leaky CreER activity led to increased thyroid volume in Tamoxifen untreated mice Unclear exactly when thyrocytes assumed malignant characteristics	[125]
Transgenic mice	Mixed	TPO-Cre; LSL-Braf^V600E^	PTC	3 weeks	Very short latency.	Very high TSH levels reported postnatally.	[126]
Transgenic mouse (Induced by Tamoxifen)	C57BL/6	Tg-CreER^T2^; LSL-Braf^V600E^	PTC	NA	Demonstrates role of Adrenomedullin2 and excessive high fat nutrition in transformation of TCs to more progressive phenotypes	Role of leptin-JAK-STAT3 activation not validated	[127]
Transgenic mouse (Induced by Tamoxifen)	Mixed C57BL/6; 129SvJae	TPOCreER; Braf^CA/+^ TPOCreER; Braf^CA/+;^ Trp53^Δex2-10/ Δex2-10^	PTC ATC	Papillary morphology visible 12 week post-induction	Recapitulates the temporal progression of BRAF-mutant PTC to ATC	Cannot induce recombination in the entire thyroid even after tamoxifen induction.	[131, 159]
Transgenic double mutant mouse (Induced by Tamoxifen)	FVB/N	Thyro::CreER; Braf^CA/+;^Pik3ca^Lat-1047R /+^	ATC	2.5 months	Development of pre-clinical modeling of combination pathway-targeted therapy	Unclear whether mutational activation of BRAF or PIK3CA can serve as strong prognostic biomarkers for thyroid cancer patient responses to pathway-targeted therapy	[132]
Conditional transgenic mice	129Sv	TPO-Cre; KRas^LSL-G12D^; Pten^Lox/Lox^	FTC	5 weeks	10% of the mice that survive for more than 12 weeks display thyroglobulin positive lung metastases.	Very aggressive; mortality within 20 weeks.	[134]
Mutation introduced in knock-out mice	C57BL/6 or 129Sv	Rb^+/-^ Nras^+/-^ Rb^+/-^ Nras^-/-^	C-cell adenomas that progress to metastatic MTC	NA	Model for metastatic disease	Pituitary tumors also observed in 47% Rb^+/-^ Nras^-/-^ animals	[155]
Transgenic mice ‘Rascal mice’	C57BL/6 X SJL	CGRP-v-Ha-ras	MTC	85%–93%; 6months—1 year	High penetrance; Model for sporadic MTC rather than MEN associated	Ras mutations are rare in human MTC	[154]
Transgenic mice	FVB/N mice	Tg-Ret/PTC1	PTC	100%; 1–6 months	First model of PTC. Exhibited nuclear features and local invasion similar to human PTC.	Lack of any metastases Elevated TSH levels.	[139]
Transgenic mice	C3H/He	Tg-Ret/PTC3 Tg-RET/PTC3p53^-/-^	PTC ATC	8 months	Model expressed human RET/PTC3 exclusively in the thyroid	Despite the large tumor burden, animals rarely developed local lymph node metastases	[140, 141]
Transgenic mice	FVB/N	Tg-(CAG-EGFP) Cre; PPFP;Pten^-/-^	TC	5 months	First mouse model of PPFP-associated TC Enables testing of PPARγ ligands as therapeutics	Histology not defined	[144]
Transgenic mice	FVB/N	Tg-STRN-ALK	PDTC	12 months	Demonstrates that STRN-ALK can drive the development of PDTC	ALK expression was not observed in mice without TSH stimulation	[145]
Transgenic mice	C57BL/6	RET2B; p18^(+/−)^ and RET2B; p18^(−/−)^	MTC	21% and 33%; 9 months	Increased incidence in double mutants		[152]
Transgenic mice	C57BL/6 x DBA2	CT/CGRP- RET^C634R^	multifocal and bilateral MTC	14 months	Model of a human hereditary neoplasia (MEN Type 2 A associated)	Long latency period MEN associated MTC account for only 25% cases	[160]
Transgenic mice	C57BL/6 x DBA2	CALC-I- RET^M918T^ (CALC-MEN2B-RET)	MTC	37%; 20–22 months	MEN Type 2 A associated	Incomplete penetrance and variable latency period	[161]
Transgenic mice	B6C3F1	Tg-TRK-T1	PTC	7 months	Model based on chromosomal rearrangements of the receptor for nerve growth factor, NTRK1 to form a fusion protein	Classic nuclear clearing typical of PCTs was lacking. None of the tumors metastasized.	[162]

The TRβ^PV/PV^ transgenic mouse model was developed via a knock-in of a dominant negative PV mutation into the thyroid hormone receptor TRβ gene locus [121]. TRβ^PV/PV^ thyroid tissues develop sequential pathological progression from extensive papillary hyperplasia to anaplasia and, ultimately, metastasis to distant organs. The disruption of the thyroid-pituitary axis in these mice results in extremely high levels of circulating thyroid stimulating hormone (TSH) which can play a pivotal role in stimulating the proliferation of thyroid cells, thereby participating in the initiation of tumorigenesis [122]. However, TC tumors in these animals are not driven by clinically relevant oncogene or tumor suppressor mutations and do not recapitulate a specific histologic subtype of human TC [121].

Genomic studies in follicular cell-derived TC revealed mutually exclusive driver mutations in BRAF or RAS. BRAF activating mutations (e.g., BRAF^V600E^) are the most common mutation in differentiated TC, seen in over 60% of sporadic PTCs [119, 123]. Several mutant BRAF-driven GEMMs of TC have been generated. Transgenic mice were generated that targeted expression of BRAF^V600E^ to thyroid cells by using the bovine thyroglobulin (Tg) promoter. Tg-BRAF^V600E^ mice develop PTC that progresses to PDTC [17]. Chakravarty et al., generated Tg-rtTA/tetO-BRAF^V600E^ mice that express BRAF^V600E^ in thyroid follicular cells in a doxycycline-inducible manner which gives rise to PTCs with short latency [124]. Moreover, conditional transgenic mice were generated with thyrocyte-specific expression of inducible Cre recombinase (CreER^T2^) under the control of the Thyroglobulin promoter (Thyro::CreER^T2^). Thyrocyte-specific induction of CreER^T2^ activity is achieved by intraperitoneal injection of Tamoxifen and mice develop well-defined PTCs after 12 months [125]. LSL-Braf^V600E^/TPO-Cre mice were established to enable endogenous expression of Braf^V600E^ in thyroid tissue which leads to the development of classical PTC with a short latency of 5 weeks [126]. Kim et al. developed a LSL-Braf^V600E^; TgCreER^T2^ mouse model for inducible thyrocyte-specific activation of Braf^V600E^, which exhibits PTC that transforms to a progressive phenotype when mice are fed a high-fat diet [127]. However, no distant metastasis are observed, suggesting that mutant BRAF alone lacks sufficient oncogenic potential and additional driver mutations are required for tumor progression.

Combination of BRAF^V600E^ or RAS-driven cancers with TERT promoter mutation or Trp53 mutation resulted in clinically more aggressive TC [128–130]. Moreover, next generation sequencing showed higher prevalence of these mutations in 60% of the analyzed PDTC patient samples as compared to only 9% of PTCs from TCGA and was also comparable to the 73% prevalence in ATC tumor samples [130]. Most of the undifferentiated TC arising from well differentiated precursors do so by acquiring additional mutations and gene alterations. GEMMs with multiple genetic abnormalities develop more aggressive types of TC. TPOCreER; Braf^CA/+^; Trp53^Δex2-10/Δex2-10^ mice harbor combined BRAF mutation and loss of p53 in thyrocytes by virtue of the thyroid peroxidase (TPO) promoter, and ultimately progress to ATC [131]. Similarly, Thyro::CreER; Braf^CA/+^; Pik3ca^H1047R/+^ conditional transgenic mice harboring the H1047R activating hotspot mutation in PIK3CA combined with BRAF mutation also develop tumors that progress to ATC [132].

Upstream of BRAF, mutations in RAS genes have been associated with 30–45% of FTCs, 30–45% of follicular variant PTC, 20–40% of PDTC, and 10–20% of ATCs [133]. The GEMM TPO-Cre; LSL-Kras^G12D^; Pten^fl/fl^ conditionally expressing mutant Kras^G12D^ and deleting the Pten gene were generated and rapid development of FTCs is observed [134]. This model was modified to generate mice with combined deletion of Pten and Trp53, TPO-Cre; Pten^fl/fl^; Trp53^fl/fl^, resulting in development of ATC [135]. Furthermore, although BRAF and RAS driver mutations are mutually exclusive in TC development, acquired resistance to BRAF inhibitors may be facilitated by KRAS or NRAS activating mutations both in the clinical and in vitro settings [136, 137]. TCs in this setting progress to PDTC/ATC. Utilizing GEMMs, such as the TPOCreER; Braf^CA/+^; Trp53^Δex2-10/Δex2-10^ mice and analyzing them in the presence of long-term targeted therapy may replicate the RAS-driven formation of resistant disease observed in patients.

Chromosomal translocations leading to the formation of fusion oncogenes play a role in the pathogenesis of many follicular cell-derived TCs and have been reported in over 15% of both PTC and FTC [119]. PTCs, particularly radiation-associated PTC, harbor the rearranged during transfection (RET) proto-oncogene and the resultant oncogenic fusion proteins are termed RET/PTCs [138]. Based on this, Jhiang et al. developed the first GEMM for TC in which the highly active bovine thyroglobulin (Tg) promoter was employed to drive transgene expression of RET/PTC1. The thyroid tumors developed in theses mice exhibited nuclear features and local invasion similar to the nuclear grooves, vesicular nuclei, and pseudo inclusions characteristic of human PTC [139]. Similarly, Powell et al. generated mice by expressing another member of the RET proto-oncogene family, RET/PTC3, exclusively in mouse thyroid [140]. Crossing Tg-RET/PTC3 mice with Trp53^−/−^ mice led to an increase in tumor burden and aggressiveness [141]. However, even after introduction of the additional Trp53 mutation, there is no evidence of local or distant metastasis. Furthermore, elevated levels of TSH are reported which is a major drawback of these models because chronic TSH stimulation is not involved in TC development in humans. The expression of the PAX8/PPARγ gene fusion is seen in ~35% of FTCs [142, 143]. To study the role of PAX8/PPARγ fusion protein (PPFP), a transgenic mouse model was generated that combined CAG promoter-driven Cre-dependent expression of PPFP with conditional homozygous deletion of Pten in the thyroid. Tg-Cre; CAG-LSL-PPFP; Pten^fl/fl^ mice develop thyroid carcinomas with distant metastasis [144]. The STRN-ALK fusions are commonly detected in PTC, PDTC and ATC, therefore transgenic Tg-STRN-ALK mice based on this chromosomal rearrangement were established. These mice exhibit thyroglobulin promoter driven thyroid-specific expression of STRN-ALK that leads to the development of PDTCs [145].

RET mutations are crucial for MTC pathogenesis in humans as over 75% of MTC are driven by RET proto-oncogene mutation, 25% of which are inherited through the germline [146]. Several distinct RET-activating point mutations within the endogenously expressed RET gene induce C-cell transformation, producing MTC [147–149]. Spontaneous activating mutations of RET were also identified in 50% of MTC bearing mice [150]. It has been attempted to establish transgenic mouse models for MEN 2-associated MTC by introducing a specific point mutation in the RET proto-oncogene [33, 34]. Thus, the development of pre-clinical models representing sporadic MTCs is highly desired. Furthermore, it has been demonstrated that the tumor penetrance can be modulated by the genetic background of the RET transgenic mice [151]. RET-induced tumors also develop in transgenic mice with loss of p18 (and p27) serving as an additional oncogenic hit required for MTC tumorigenesis [152]. Harvey et al. were the pioneers in generating GEMMs of endocrine tumor types with heterozygous deletion of Rb and Trp53 (Rb^+/−^; p53^+/−^). However, in addition to MTC, these mice develop pancreatic islet cell carcinomas, pituitary adenomas, lymphomas and sarcomas [153]. The neuroendocrine specific calcitonin gene-related peptide (CGRP) promoter was used to express v-Ha-ras oncogene in the thyroid C-cells in mice of the C57BL/6 x SJL strain. Tumors develop with high penetrance and secrete calcitonin [154]. Another model with NRAS mutation in Rb knock-out mice was established which develop metastatic MTCs. However, *RAS* mutations are rare in clinical cases and hence this model is more representative of the human MTC phenotype but not the genotype [155].

## Conclusions

A plethora of GEMMs recapitulating both squamous and glandular HNCs are now available for investigating the molecular pathways involved in tumor development and progression. Numerous efforts have improved and enriched the repertoire of HNC GEMMs to exhibit the various histologic subtype features of this diverse group of cancers, thereby enabling the selection of a suitable model for evaluating personalized cancer therapies. However, genetic alterations are engineered and present in all cells of a certain type, and GEMMs therefore may not completely represent the tumor heterogeneity and complexity found in human HNCs. It is also difficult to recapitulate metastatic progression, since animals tend to become moribund due to large burden of the primary tumor and must be euthanized often at early stages of disease. Furthermore, generating GEMMs remains highly time and labor intensive, even with the advent of CRISPR-based methodologies, and tumor development is extremely variable due to differences in penetrance and latency between models. Despite these limitations, GEMMs serve as a robust tool for understanding basic tumor biology and for preclinical testing of novel therapeutic modalities. Thus, continued development and refinement of GEMMs that reproducibly display key features of the human malignancies are required to enable investigators to evaluate the local and systemic responses to next generation treatment.
